# Measurement of $\psi {(2S)}$ polarisation in $pp$ collisions at $\sqrt{s}$ = 7 TeV

**DOI:** 10.1140/epjc/s10052-014-2872-9

**Published:** 2014-05-20

**Authors:** The LHCb Collaboration, R. Aaij, B. Adeva, M. Adinolfi, A. Affolder, Z. Ajaltouni, J. Albrecht, F. Alessio, M. Alexander, S. Ali, G. Alkhazov, P. Alvarez Cartelle, A. A. Alves, S. Amato, S. Amerio, Y. Amhis, L. An, L. Anderlini, J. Anderson, R. Andreassen, M. Andreotti, J. E. Andrews, R. B. Appleby, O. Aquines Gutierrez, F. Archilli, A. Artamonov, M. Artuso, E. Aslanides, G. Auriemma, M. Baalouch, S. Bachmann, J. J. Back, A. Badalov, V. Balagura, W. Baldini, R. J. Barlow, C. Barschel, S. Barsuk, W. Barter, V. Batozskaya, Th. Bauer, A. Bay, J. Beddow, F. Bedeschi, I. Bediaga, S. Belogurov, K. Belous, I. Belyaev, E. Ben-Haim, G. Bencivenni, S. Benson, J. Benton, A. Berezhnoy, R. Bernet, M.-O. Bettler, M. van Beuzekom, A. Bien, S. Bifani, T. Bird, A. Bizzeti, P. M. Bjørnstad, T. Blake, F. Blanc, J. Blouw, S. Blusk, V. Bocci, A. Bondar, N. Bondar, W. Bonivento, S. Borghi, A. Borgia, M. Borsato, T. J. V. Bowcock, E. Bowen, C. Bozzi, T. Brambach, J. van den Brand, J. Bressieux, D. Brett, M. Britsch, T. Britton, N. H. Brook, H. Brown, A. Bursche, G. Busetto, J. Buytaert, S. Cadeddu, R. Calabrese, O. Callot, M. Calvi, M. Calvo Gomez, A. Camboni, P. Campana, D. Campora Perez, A. Carbone, G. Carboni, R. Cardinale, A. Cardini, H. Carranza-Mejia, L. Carson, K. Carvalho Akiba, G. Casse, L. Cassina, L. Castillo Garcia, M. Cattaneo, Ch. Cauet, R. Cenci, M. Charles, Ph. Charpentier, S.-F. Cheung, N. Chiapolini, M. Chrzaszcz, K. Ciba, X. Cid Vidal, G. Ciezarek, P. E. L. Clarke, M. Clemencic, H. V. Cliff, J. Closier, C. Coca, V. Coco, J. Cogan, E. Cogneras, P. Collins, A. Comerma-Montells, A. Contu, A. Cook, M. Coombes, S. Coquereau, G. Corti, M. Corvo, I. Counts, B. Couturier, G. A. Cowan, D. C. Craik, M. Cruz Torres, S. Cunliffe, R. Currie, C. D’Ambrosio, J. Dalseno, P. David, P. N. Y. David, A. Davis, K. De Bruyn, S. De Capua, M. De Cian, J. M. De Miranda, L. De Paula, W. De Silva, P. De Simone, D. Decamp, M. Deckenhoff, L. Del Buono, N. Déléage, D. Derkach, O. Deschamps, F. Dettori, A. Di Canto, H. Dijkstra, S. Donleavy, F. Dordei, M. Dorigo, A. Dosil Suárez, D. Dossett, A. Dovbnya, F. Dupertuis, P. Durante, R. Dzhelyadin, A. Dziurda, A. Dzyuba, S. Easo, U. Egede, V. Egorychev, S. Eidelman, S. Eisenhardt, U. Eitschberger, R. Ekelhof, L. Eklund, I. El Rifai, Ch. Elsasser, S. Esen, T. Evans, A. Falabella, C. Färber, C. Farinelli, S. Farry, D. Ferguson, V. Fernandez Albor, F. Ferreira Rodrigues, M. Ferro-Luzzi, S. Filippov, M. Fiore, M. Fiorini, M. Firlej, C. Fitzpatrick, T. Fiutowski, M. Fontana, F. Fontanelli, R. Forty, O. Francisco, M. Frank, C. Frei, M. Frosini, J. Fu, E. Furfaro, A. Gallas Torreira, D. Galli, M. Gandelman, P. Gandini, Y. Gao, J. Garofoli, J. Garra Tico, L. Garrido, C. Gaspar, R. Gauld, L. Gavardi, E. Gersabeck, M. Gersabeck, T. Gershon, Ph. Ghez, A. Gianelle, S. Giani, V. Gibson, L. Giubega, V. V. Gligorov, C. Göbel, D. Golubkov, A. Golutvin, A. Gomes, H. Gordon, C. Gotti, M. Grabalosa Gándara, R. Graciani Diaz, L. A. Granado Cardoso, E. Graugés, G. Graziani, A. Grecu, E. Greening, S. Gregson, P. Griffith, L. Grillo, O. Grünberg, B. Gui, E. Gushchin, Yu. Guz, T. Gys, C. Hadjivasiliou, G. Haefeli, C. Haen, S. C. Haines, S. Hall, B. Hamilton, T. Hampson, X. Han, S. Hansmann-Menzemer, N. Harnew, S. T. Harnew, J. Harrison, T. Hartmann, J. He, T. Head, V. Heijne, K. Hennessy, P. Henrard, L. Henry, J. A. Hernando Morata, E. van Herwijnen, M. Heß, A. Hicheur, D. Hill, M. Hoballah, C. Hombach, W. Hulsbergen, P. Hunt, N. Hussain, D. Hutchcroft, D. Hynds, V. Iakovenko, M. Idzik, P. Ilten, R. Jacobsson, A. Jaeger, J. Jalocha, E. Jans, P. Jaton, A. Jawahery, M. Jezabek, F. Jing, M. John, D. Johnson, C. R. Jones, C. Joram, B. Jost, N. Jurik, M. Kaballo, S. Kandybei, W. Kanso, M. Karacson, T. M. Karbach, M. Kelsey, I. R. Kenyon, T. Ketel, B. Khanji, C. Khurewathanakul, S. Klaver, O. Kochebina, M. Kolpin, I. Komarov, R. F. Koopman, P. Koppenburg, M. Korolev, A. Kozlinskiy, L. Kravchuk, K. Kreplin, M. Kreps, G. Krocker, P. Krokovny, F. Kruse, M. Kucharczyk, V. Kudryavtsev, K. Kurek, T. Kvaratskheliya, V. N. La Thi, D. Lacarrere, G. Lafferty, A. Lai, D. Lambert, R. W. Lambert, E. Lanciotti, G. Lanfranchi, C. Langenbruch, T. Latham, C. Lazzeroni, R. Le Gac, J. van Leerdam, J.-P. Lees, R. Lefèvre, A. Leflat, J. Lefrançois, S. Leo, O. Leroy, T. Lesiak, B. Leverington, Y. Li, M. Liles, R. Lindner, C. Linn, F. Lionetto, B. Liu, G. Liu, S. Lohn, I. Longstaff, I. Longstaff, J. H. Lopes, N. Lopez-March, P. Lowdon, H. Lu, D. Lucchesi, J. Luisier, H. Luo, A. Lupato, E. Luppi, O. Lupton, F. Machefert, I. V. Machikhiliyan, F. Maciuc, O. Maev, S. Malde, G. Manca, G. Mancinelli, M. Manzali, J. Maratas, J. F. Marchand, U. Marconi, P. Marino, R. Märki, J. Marks, G. Martellotti, A. Martens, A. Martín Sánchez, M. Martinelli, D. Martinez Santos, F. Martinez Vidal, D. Martins Tostes, A. Massafferri, R. Matev, Z. Mathe, C. Matteuzzi, A. Mazurov, M. McCann, J. McCarthy, A. McNab, R. McNulty, B. McSkelly, B. Meadows, F. Meier, M. Meissner, M. Merk, D. A. Milanes, M.-N. Minard, J. Molina Rodriguez, S. Monteil, D. Moran, M. Morandin, P. Morawski, A. Mordà, M. J. Morello, J. Moron, R. Mountain, F. Muheim, K. Müller, R. Muresan, B. Muster, P. Naik, T. Nakada, R. Nandakumar, I. Nasteva, M. Needham, N. Neri, S. Neubert, N. Neufeld, M. Neuner, A. D. Nguyen, T. D. Nguyen, C. Nguyen-Mau, M. Nicol, V. Niess, R. Niet, N. Nikitin, T. Nikodem, A. Novoselov, A. Oblakowska-Mucha, V. Obraztsov, S. Oggero, S. Ogilvy, O. Okhrimenko, R. Oldeman, G. Onderwater, M. Orlandea, J. M. Otalora Goicochea, P. Owen, A. Oyanguren, B. K. Pal, A. Palano, F. Palombo, M. Palutan, J. Panman, A. Papanestis, M. Pappagallo, C. Parkes, C. J. Parkinson, G. Passaleva, G. D. Patel, M. Patel, C. Patrignani, A. Pazos Alvarez, A. Pearce, A. Pellegrino, G. Penso, M. Pepe Altarelli, S. Perazzini, E. Perez Trigo, P. Perret, M. Perrin-Terrin, L. Pescatore, E. Pesen, K. Petridis, A. Petrolini, E. Picatoste Olloqui, B. Pietrzyk, T. Pilař, D. Pinci, A. Pistone, S. Playfer, M. Plo Casasus, F. Polci, G. Polok, A. Poluektov, E. Polycarpo, A. Popov, D. Popov, B. Popovici, C. Potterat, A. Powell, J. Prisciandaro, A. Pritchard, C. Prouve, V. Pugatch, A. Puig Navarro, G. Punzi, W. Qian, B. Rachwal, J. H. Rademacker, B. Rakotomiaramanana, M. Rama, M. S. Rangel, I. Raniuk, N. Rauschmayr, G. Raven, S. Redford, S. Reichert, M. M. Reid, A. C. dos Reis, S. Ricciardi, A. Richards, K. Rinnert, V. Rives Molina, D. A. Roa Romero, P. Robbe, A. B. Rodrigues, E. Rodrigues, P. Rodriguez Perez, S. Roiser, V. Romanovsky, A. Romero Vidal, M. Rotondo, J. Rouvinet, T. Ruf, F. Ruffini, H. Ruiz, P. Ruiz Valls, G. Sabatino, J. J. Saborido Silva, N. Sagidova, P. Sail, B. Saitta, V. Salustino Guimaraes, C. Sanchez Mayordomo, B. Sanmartin Sedes, R. Santacesaria, C. Santamarina Rios, E. Santovetti, M. Sapunov, A. Sarti, C. Satriano, A. Satta, M. Savrie, D. Savrina, M. Schiller, H. Schindler, M. Schlupp, M. Schmelling, B. Schmidt, O. Schneider, A. Schopper, M.-H. Schune, R. Schwemmer, B. Sciascia, A. Sciubba, M. Seco, A. Semennikov, K. Senderowska, I. Sepp, N. Serra, J. Serrano, L. Sestini, P. Seyfert, M. Shapkin, I. Shapoval, Y. Shcheglov, T. Shears, L. Shekhtman, V. Shevchenko, A. Shires, R. Silva Coutinho, G. Simi, M. Sirendi, N. Skidmore, T. Skwarnicki, N. A. Smith, E. Smith, E. Smith, J. Smith, M. Smith, H. Snoek, M. D. Sokoloff, F. J. P. Soler, F. Soomro, D. Souza, B. Souza De Paula, B. Spaan, A. Sparkes, F. Spinella, P. Spradlin, F. Stagni, S. Stahl, O. Steinkamp, O. Stenyakin, S. Stevenson, S. Stoica, S. Stone, B. Storaci, S. Stracka, M. Straticiuc, U. Straumann, R. Stroili, V. K. Subbiah, L. Sun, W. Sutcliffe, K. Swientek, S. Swientek, V. Syropoulos, M. Szczekowski, P. Szczypka, D. Szilard, T. Szumlak, S. T’Jampens, M. Teklishyn, G. Tellarini, E. Teodorescu, F. Teubert, C. Thomas, E. Thomas, J. van Tilburg, V. Tisserand, M. Tobin, S. Tolk, L. Tomassetti, D. Tonelli, S. Topp-Joergensen, N. Torr, E. Tournefier, S. Tourneur, M. T. Tran, M. Tresch, A. Tsaregorodtsev, P. Tsopelas, N. Tuning, M. Ubeda Garcia, A. Ukleja, A. Ustyuzhanin, U. Uwer, V. Vagnoni, G. Valenti, A. Vallier, R. Vazquez Gomez, P. Vazquez Regueiro, C. Vázquez Sierra, S. Vecchi, J. J. Velthuis, M. Veltri, G. Veneziano, M. Vesterinen, B. Viaud, D. Vieira, M. Vieites Diaz, X. Vilasis-Cardona, A. Vollhardt, D. Volyanskyy, D. Voong, A. Vorobyev, V. Vorobyev, C. Voß, H. Voss, J. A. de Vries, R. Waldi, C. Wallace, R. Wallace, J. Walsh, S. Wandernoth, J. Wang, D. R. Ward, N. K. Watson, A. D. Webber, D. Websdale, M. Whitehead, J. Wicht, D. Wiedner, L. Wiggers, G. Wilkinson, M. P. Williams, M. Williams, F. F. Wilson, J. Wimberley, J. Wishahi, W. Wislicki, M. Witek, G. Wormser, S. A. Wotton, S. Wright, S. Wu, K. Wyllie, Y. Xie, Z. Xing, Z. Xu, Z. Yang, X. Yuan, O. Yushchenko, M. Zangoli, M. Zavertyaev, F. Zhang, L. Zhang, W. C. Zhang, Y. Zhang, A. Zhelezov, A. Zhokhov, L. Zhong, A. Zvyagin

**Affiliations:** 1 Centro Brasileiro de Pesquisas Físicas (CBPF), Rio de Janeiro, Brazil; 2 Universidade Federal do Rio de Janeiro (UFRJ), Rio de Janeiro, Brazil; 3 Center for High Energy Physics, Tsinghua University, Beijing, China; 4 LAPP, Université de Savoie, CNRS/IN2P3, Annecy-Le-Vieux, France; 5 Clermont Université, Université Blaise Pascal, CNRS/IN2P3, LPC, Clermont-Ferrand, France; 6 CPPM, Aix-Marseille Université, CNRS/IN2P3, Marseille, France; 7 LAL, Université Paris-Sud, CNRS/IN2P3, Orsay, France; 8 LPNHE, Université Pierre et Marie Curie, Université Paris Diderot, CNRS/IN2P3, Paris, France; 9 Fakultät Physik, Technische Universität Dortmund, Dortmund, Germany; 10 Max-Planck-Institut für Kernphysik (MPIK), Heidelberg, Germany; 11 Physikalisches Institut, Ruprecht-Karls-Universität Heidelberg, Heidelberg, Germany; 12 School of Physics, University College Dublin, Dublin, Ireland; 13 Sezione INFN di Bari, Bari, Italy; 14 Sezione INFN di Bologna, Bologna, Italy; 15 Sezione INFN di Cagliari, Cagliari, Italy; 16 Sezione INFN di Ferrara, Ferrara, Italy; 17 Sezione INFN di Firenze, Florence, Italy; 18 Laboratori Nazionali dell’INFN di Frascati, Frascati, Italy; 19 Sezione INFN di Genova, Genova, Italy; 20 Sezione INFN di Milano Bicocca, Milan, Italy; 21 Sezione INFN di Milano, Milan, Italy; 22 Sezione INFN di Padova, Padua, Italy; 23 Sezione INFN di Pisa, Pisa, Italy; 24 Sezione INFN di Roma Tor Vergata, Rome, Italy; 25 Sezione INFN di Roma La Sapienza, Rome, Italy; 26 Henryk Niewodniczanski Institute of Nuclear Physics Polish Academy of Sciences, Kraków, Poland; 27 Faculty of Physics and Applied Computer Science, AGH-University of Science and Technology, Kraków, Poland; 28 National Center for Nuclear Research (NCBJ), Warsaw, Poland; 29 Horia Hulubei National Institute of Physics and Nuclear Engineering, Bucharest-Magurele, Romania; 30 Petersburg Nuclear Physics Institute (PNPI), Gatchina, Russia; 31 Institute of Theoretical and Experimental Physics (ITEP), Moscow, Russia; 32 Institute of Nuclear Physics, Moscow State University (SINP MSU), Moscow, Russia; 33 Institute for Nuclear Research of the Russian Academy of Sciences (INR RAN), Moscow, Russia; 34 Budker Institute of Nuclear Physics (SB RAS), Novosibirsk State University, Novosibirsk, Russia; 35 Institute for High Energy Physics (IHEP), Protvino, Russia; 36 Universitat de Barcelona, Barcelona, Spain; 37 Universidad de Santiago de Compostela, Santiago de Compostela, Spain; 38 European Organization for Nuclear Research (CERN), Geneva, Switzerland; 39 Ecole Polytechnique Fédérale de Lausanne (EPFL), Lausanne, Switzerland; 40 Physik-Institut, Universität Zürich, Zurich, Switzerland; 41 Nikhef National Institute for Subatomic Physics, Amsterdam, The Netherlands; 42 Nikhef National Institute for Subatomic Physics, VU University Amsterdam, Amsterdam, The Netherlands; 43 NSC Kharkiv Institute of Physics and Technology (NSC KIPT), Kharkiv, Ukraine; 44 Institute for Nuclear Research of the National Academy of Sciences (KINR), Kiev, Ukraine; 45 University of Birmingham, Birmingham, UK; 46 H.H. Wills Physics Laboratory, University of Bristol, Bristol, UK; 47 Cavendish Laboratory, University of Cambridge, Cambridge, UK; 48 Department of Physics, University of Warwick, Coventry, UK; 49 STFC Rutherford Appleton Laboratory, Didcot, UK; 50 School of Physics and Astronomy, University of Edinburgh, Edinburgh, UK; 51 School of Physics and Astronomy, University of Glasgow, Glasgow, UK; 52 Oliver Lodge Laboratory, University of Liverpool, Liverpool, UK; 53 Imperial College London, London, UK; 54 School of Physics and Astronomy, University of Manchester, Manchester, UK; 55 Department of Physics, University of Oxford, Oxford, UK; 56 Massachusetts Institute of Technology, Cambridge, MA USA; 57 University of Cincinnati, Cincinnati, OH USA; 58 University of Maryland, College Park, MD USA; 59 Syracuse University, Syracuse, NY USA; 60 Pontifícia Universidade Católica do Rio de Janeiro (PUC-Rio), Rio de Janeiro, Brazil; 61 Institute of Particle Physics, Central China Normal University, Wuhan, Hubei China; 62 Institut für Physik, Universität Rostock, Rostock, Germany; 63 National Research Centre Kurchatov Institute, Moscow, Russia; 64 Instituto de Fisica Corpuscular (IFIC), Universitat de Valencia-CSIC, Valencia, Spain; 65 KVI, University of Groningen, Groningen, The Netherlands; 66 Celal Bayar University, Manisa, Turkey; 67 CERN, 1211 Geneva 23, Switzerland

## Abstract

The polarisation of prompt $\psi {(2S)}$ mesons is measured by performing an angular analysis of $\psi {(2S)} \!\rightarrow \mu ^+\mu ^-$ decays using proton-proton collision data, corresponding to an integrated luminosity of 1.0$\,\text{ fb }^{-1}$, collected by the LHCb detector at a centre-of-mass energy of 7 TeV. The polarisation is measured in bins of transverse momentum $p_\mathrm{T}$ and rapidity $y$ in the kinematic region $3.5< p_\mathrm{T} <15{\mathrm {\,GeV\!/}c}$ and $2.0<y<4.5$, and is compared to theoretical models. No significant polarisation is observed.

## Introduction

Measurements of the heavy quarkonium production in hadron collisions can be used to test predictions of quantum chromodynamics (QCD) in the perturbative and non-perturbative regimes. Several theoretical models have been developed within the framework of QCD to describe the quarkonium production cross-section and polarisation as functions of the quarkonium transverse momentum, $p_\mathrm{T}$, but none can simultaneously describe both of them [1]. Heavy quarkonia can be produced in three ways in $pp$ collisions: directly in the hard scattering, through feed-down from higher-mass quarkonia states, or via the decay of $b$ hadrons, with the first two of these being referred to as prompt production. In the case of $\psi {(2S)}$ mesons, the contribution from feed-down is negligible, allowing a straightforward comparison between measurements of prompt production and predictions for direct contributions.

The $\psi {(2S)}$ meson has spin, parity and charge-parity quantum numbers, $J^{\mathrm {PC}}=1^{--}$ and its polarisation can be determined by studying the angular distribution of muons in the $\psi {(2S)} \!\rightarrow \mu ^+ \mu ^-$ decays [2, 3]. The distribution is described by

1 $$\begin{aligned} \frac{d^2N}{d\cos \theta \;d\phi }(\lambda _\theta ,\lambda _{\theta \phi },\lambda _\phi ) \propto 1+\lambda _\theta \cos ^2\!\theta \nonumber \\ + \lambda _{\theta \phi }\sin 2\theta \cos \phi + \lambda _\phi \sin ^2\!\theta \cos 2\phi , \end{aligned}$$

where $\theta$ and $\phi$ are the polar and azimuthal angles of the $\mu ^+$ direction in the rest frame of the $\psi {(2S)}$ meson, respectively, and $\lambda _\theta ,\lambda _{\theta \phi }$ and $\lambda _\phi$ are the polarisation parameters to be determined from the data. The case of $(\lambda _{\theta }, \lambda _{\theta \phi }, \lambda _{\phi } ) = (1,\,0,\,0)$ or $(-1\,,0,\,0)$ corresponds to full transverse or longitudinal polarisation, respectively, while $(\lambda _\theta ,\lambda _{\theta \phi },\lambda _\phi )=(0,\,0,\,0)$ corresponds to the unpolarised state.^1^ In this study of the $\psi {(2S)}$ polarisation, two choices of polarisation frame are used. These have a common definition of the $Y$-axis, taken to be the normal to the production plane, which is formed by the momentum of the $\psi {(2S)}$ meson and the beam axis in the rest frame of the colliding LHC protons. The helicity frame [4] uses the $\psi {(2S)}$ momentum as the $Z$-axis. In the Collins-Soper frame [5] the $Z$-axis is chosen to be the bisector of the angle between the two incoming proton beams in the rest frame of the $\psi {(2S)}$ meson. In both frames, the $X$-axis is defined to complete a right-handed Cartesian coordinate system. The commonly used frame-invariant variable $\lambda _\mathrm {inv}$ (see [6, 7]) is defined as

2 $$\begin{aligned} \lambda _{\mathrm {inv}} = \frac{\lambda _\theta +3\lambda _\phi }{1-\lambda _\phi }. \end{aligned}$$

Two classes of theoretical models are compared with the measurements in this paper: the colour-singlet model (CSM) [8] and non-relativistic QCD (NRQCD) [9–14], at next-to-leading order (NLO). In the high-$p_\mathrm{T}$ region, where the quarkonium transverse momentum is much larger than its mass (in natural units), the CSM underestimates significantly the measured prompt ${J /\psi }$ and $\psi {(2S)}$ production cross-sections [15–17], while the NRQCD model provides a good description of the $p_\mathrm{T}$-dependent ${J /\psi }$ and $\psi {(2S)}$ cross-sections measured by LHCb  [16, 17] and CMS  [18]. The CSM predicts large longitudinal polarisation for ${J /\psi }$ and $\psi {(2S)}$ mesons. On the other hand, in the NRQCD model, where quarkonium production is dominated by the gluon fragmentation process in the high-$p_\mathrm{T}$ region, the gluon is almost on-shell, leading to predictions of large transverse polarisations [11]. Precise measurements of the ${J /\psi }$ polarisation at both the Tevatron [19] and the LHC [20–22], which show no significant longitudinal or transverse polarisations, do not support either the CSM or NRQCD predictions.

The prompt $\psi {(2S)}$ polarisation has been measured by the CDF experiment [19] in $p\overline{p}$ collisions at $\sqrt{s}=1.96\,\mathrm{TeV}$, and by the CMS experiment [21] in $pp$ collisions at $\sqrt{s}=7\,\mathrm{TeV}$, using the $\psi {(2S)} \!\rightarrow \mu ^+ \mu ^-$ decay. The CDF (CMS) measurement used $\psi {(2S)}$ mesons in the kinematic range $5<p_\mathrm{T} <30{\mathrm {\,GeV\!/}c}$ ($14<p_\mathrm{T} <50{\mathrm {\,GeV\!/}c}$) and rapidity $|y|<0.6$ ($|y|<1.5$). The CDF result for $p_\mathrm{T} >10{\mathrm {\,GeV\!/}c}$ is in strong disagreement with the NRQCD prediction of large transverse polarisation. At CMS, no evidence of large transverse or longitudinal $\psi {(2S)}$ polarisation has been observed.

This paper presents the measurement of the prompt $\psi {(2S)}$ polarisation in $pp$ collisions at $\sqrt{s} =7\,\mathrm{TeV}$, using data corresponding to an integrated luminosity of 1$\,\text{ fb }^{-1}$, from $\psi {(2S)} \!\rightarrow \mu ^+ \mu ^-$ decays. The $\psi {(2S)}$ polarisation parameters are determined using unbinned maximum likelihood fits to the two-dimensional angular distribution of the $\mu ^+$ in the helicity and Collins-Soper frames. The measurement is performed in the $\psi {(2S)}$ kinematic range $3.5<p_\mathrm{T} <15{\mathrm {\,GeV\!/}c}$ and $2.0<y<4.5$.

## LHCb detector and data sample

The LHCb detector [23] is a single-arm forward spectrometer covering the pseudorapidity range $$2<\eta <5$$, designed for the study of particles containing $$b $$ or $$c $$ quarks. The detector includes a high-precision tracking system consisting of a silicon-strip vertex detector surrounding the $$pp$$ interaction region, a large-area silicon-strip detector located upstream of a dipole magnet with a bending power of about $$4\mathrm{\,Tm}$$, and three stations of silicon-strip detectors and straw drift tubes placed downstream. The combined tracking system provides a momentum measurement with relative uncertainty that varies from 0.4 % at 5$${\mathrm {\,GeV\!/}c}$$ to 0.6 % at 100$${\mathrm {\,GeV\!/}c}$$, and impact parameter resolution of 20$${\,\upmu \mathrm m}$$ for tracks with large transverse momentum. Different types of charged hadrons are distinguished by information from two ring-imaging Cherenkov detectors [24]. Photon, electron and hadron candidates are identified by a calorimeter system consisting of scintillating-pad and preshower detectors, an electromagnetic calorimeter and a hadronic calorimeter. Muons are identified by a system composed of alternating layers of iron and multiwire proportional chambers [25].

The trigger [26] consists of a hardware stage, based on information from the calorimeter and muon systems, followed by a software stage, which applies full event reconstruction. The hardware trigger requires the $p_\mathrm{T}$ of one muon candidate to be larger than $1.48{\mathrm {\,GeV\!/}c}$, or the product of the transverse momenta of two muon candidates to be larger than $1.68{\,(\mathrm {GeV\!/}c)^2}$. In a first stage of the software trigger, two oppositely charged muon candidates with $p_\mathrm{T} >0.5{\mathrm {\,GeV\!/}c}$ and momentum $p>6{\mathrm {\,GeV\!/}c}$ are selected and their invariant mass is required to be greater than $2.7{\mathrm {\,GeV\!/}c^2}$. In a second stage of the software trigger, two muon candidates consistent with originating from a $\psi {(2S)}$ decay are chosen and additional criteria are applied to refine the sample of the $\psi {(2S)}$ candidates as follows. The invariant mass of the candidate is required to be consistent with the known $\psi {(2S)}$ mass [27] and, for 0.7$\,\text{ fb }^{-1}$ of data, the $p_\mathrm{T}$ of the candidate is required to be greater than 3.5${\mathrm {\,GeV\!/}c}$.

In the simulation, $pp$ collisions are generated using Pythia  [28] with a specific LHCb configuration [29]. Decays of hadronic particles are described by EvtGen  [30], in which final state radiation is generated using Photos  [31]. The interaction of the generated particles with the detector and its response are implemented using the Geant4 toolkit [32, 33] as described in Ref. [34]. The prompt charmonium production is simulated in Pythia according to the leading order colour-singlet and colour-octet mechanisms [29, 35], and the charmonium is generated without polarisation.

## Event selection

The $\psi {(2S)}$ candidates are reconstructed from pairs of good quality, oppositely charged particles that originate from a common vertex. The $\chi ^2$ probability of the vertex fit must be larger than 0.5 %. The transverse momentum of each particle is required to be greater than $1{\mathrm {\,GeV\!/}c}$. Both tracks must also be consistent with the muon hypothesis. As in the measurement of ${J /\psi }$ polarisation [22], the significance $S_\tau$, which is defined as the reconstructed pseudo-decay time $\tau$ divided by its uncertainty, is used to distinguish between prompt $\psi {(2S)}$ mesons and those from $b$-hadron decays. The pseudo-decay time $\tau$ is defined as [17]

3 $$\begin{aligned} \tau \equiv \frac{(z_{\psi {(2S)}}-z_{\mathrm {PV}})\cdot {}M_{\psi {(2S)}}}{p_z}, \end{aligned}$$

where $z_{\psi {(2S)}}$ ($z_{\mathrm {PV}})$ is the position of the $\psi {(2S)}$ decay vertex (the associated primary vertex) in the $z$-direction, $M_{\psi {(2S)}}$ is the known $\psi {(2S)}$ mass, and $p_z$ is the measured $z$ component of the $\psi {(2S)}$ momentum in the centre-of-mass frame of the $pp$ collision. The $z$-axis of the LHCb coordinate system is defined as the beam direction in the LHCb detector region. The $\psi {(2S)}$ mesons from $b$-hadron decays tend to have large values of $S_\tau$. The requirement $S_\tau <4$ reduces the fraction of the selected non-prompt $\psi {(2S)}$ mesons from about 20 to 3 % [17, 22].

**Fig. 1:**
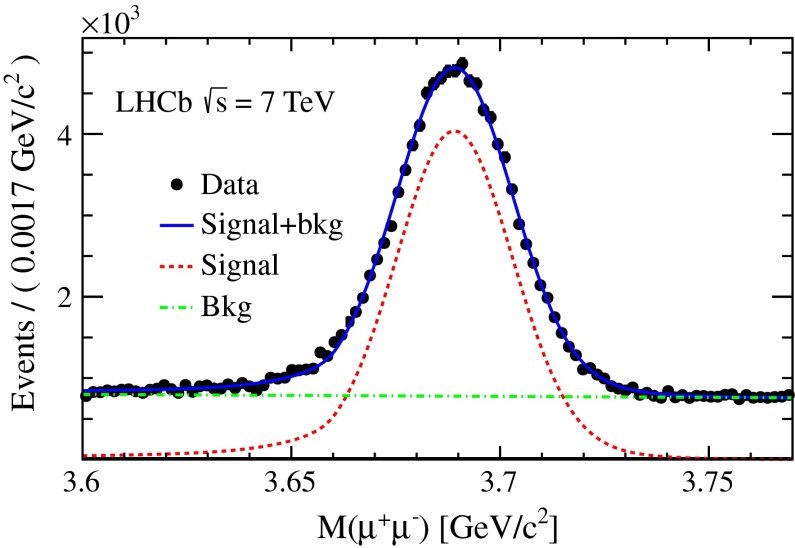
Invariant mass distribution of $\psi {(2S)}$ candidates in the kinematic region $5<p_\mathrm{T} <7{\mathrm {\,GeV\!/}c}$ and $2.5<y<3.0$. The *solid blue line* is the total fit function, the *dot-dashed green line* represents the linear background function and the *red dashed line* is the combination of the two CB functions

The analysis is performed in five $p_\mathrm{T}$ and five $y$ bins of the $\psi {(2S)}$ meson. As an example, the invariant mass distribution of $\psi {(2S)}$ candidates for $5<p_\mathrm{T} <7{\mathrm {\,GeV\!/}c}$ and $3.0<y<3.5$ is shown in Fig. 1. In each kinematic bin, the mass distribution is fitted with a combination of two Crystal Ball (CB) functions [36] with a common peak position for the signal and a linear function for the combinatorial background. The relative fractions of the narrower and broader CB functions are fixed to 0.9 and 0.1, respectively, determined from simulation.

Using the results of the fit to the mass distribution, the *sWeight* $w_i$ for each candidate $i$ to be signal is computed by means of the *sPlot* technique [37]. The correlation between the invariant mass of the $\psi {(2S)}$ candidates and the muon angular variables is found to be negligible, and the *sWeights* are used to subtract the background from the angular distribution.

## Polarisation fit

**Fig. 2:**
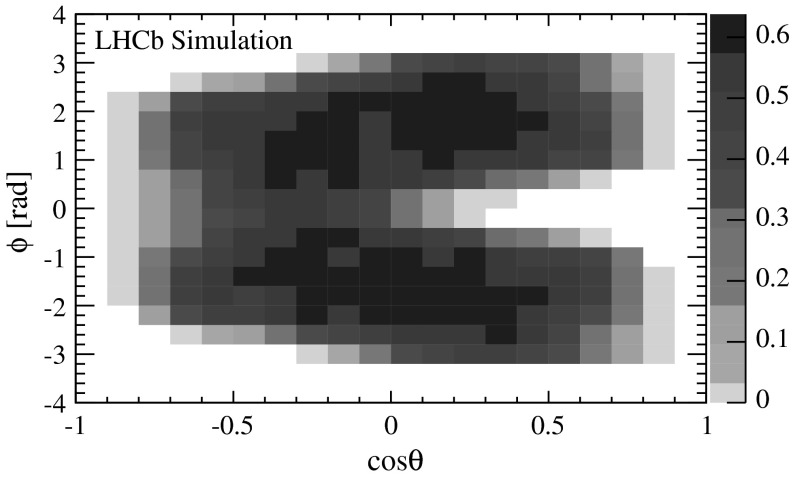
Detection efficiency in arbitrary units as a function of $\cos \theta$ and $\phi$ in the helicity frame for $\psi {(2S)}$ mesons in the range $5<p_\mathrm{T} <7{\mathrm {\,GeV\!/}c}$ and $2.5<y<3.0$

The polarisation parameters are determined from a fit to the ($\cos \theta ,\phi$) angular distribution of the $\psi {(2S)} \!\rightarrow \mu ^+ \mu ^-$  signal candidates in each kinematic bin of the $\psi {(2S)}$ meson independently. The angular distribution described by Eq. 1 is modified by the detection efficiency $\epsilon$, which varies as a function of the angular variables ($\cos \theta ,\phi$). In each kinematic bin, $\epsilon$ is obtained from a sample of simulated unpolarised $\psi {(2S)} \!\rightarrow \mu ^+ \mu ^-$ decays, where $\cos \theta$ and $\phi$ are generated according to uniform distributions. As an example, Fig. 2 shows the efficiency in the helicity frame for $\psi {(2S)}$ candidates in the kinematic bin $5<p_\mathrm{T} <7{\mathrm {\,GeV\!/}c}$ and $2.5<y<3.0$. The typical absolute efficiency is about 10 %. For smaller (larger) $p_\mathrm{T}$ and $y$ values, the coverage of the reconstructed muon angular variables is narrower (broader). In the regions $\vert \cos \theta \vert \approx 1$, and $\vert \phi \vert \approx 0$ or $\pi$, the efficiency is lower because one of the two muons is likely to escape the LHCb detector acceptance.

Combining the angular distribution given in Eq. 1 with the efficiency, the logarithm of the likelihood function [38], in each $p_\mathrm{T}$ and $y$ bin, is defined as

4 $$\begin{aligned}&\ln L\nonumber \\&\quad =\alpha \sum ^{N_{\mathrm {tot}}}_{i=1}w_i\times \ln \left[ \frac{P(\cos \theta _{i},\phi _{i}\vert \lambda _{\theta }, \lambda _{\theta \phi }, \lambda _{\phi })\;\epsilon (\cos \theta _{i},\phi _{i})}{N(\lambda _{\theta }, \lambda _{\theta \phi }, \lambda _{\phi })}\right] , \nonumber \\ \end{aligned}$$

where $P(\cos \theta _{i},\phi _{i}\vert \lambda _{\theta },\lambda _{\theta \phi }, \lambda _{\phi }) \equiv 1+\lambda _\theta \cos ^2 \theta _{i} + \lambda _{\theta \phi }\sin 2\theta _{i} \cos \phi _{i} + \lambda _\phi \sin ^2 \theta _{i} \cos 2\phi _{i}$, $w_i$ is the *sWeight*, and $N_\mathrm {tot}$ is the number of $\psi {(2S)}$ candidates in the data. The global factor $\alpha \equiv \sum _{i=1}^{N_\mathrm {tot}}w_i/\sum _{i=1}^{N_\mathrm {tot}}w_i^2$ is introduced to estimate correctly the statistical uncertainty for the weighted likelihood function. The normalisation $N(\lambda _{\theta }, \lambda _{\theta \phi }, \lambda _{\phi })$ is defined as

5 $$\begin{aligned} N(\lambda _{\theta },\lambda _{\theta \phi },\lambda _{\phi })&= \int d\Omega P(\cos \theta ,\phi \vert \lambda _{\theta }, \lambda _{\theta \phi }, \lambda _{\phi })\nonumber \\&\times \epsilon (\cos \theta ,\phi ) \nonumber \\&\approx C\sum _{j=1}^{M_\mathrm {tot}} P(\cos \theta _j,\phi _j\vert \lambda _{\theta }, \lambda _{\theta \phi }, \lambda _{\phi }), \end{aligned}$$

where the sum extends over the $M_\mathrm {tot}$ candidates in the simulated and reconstructed sample and $C$ is a constant factor. The last equality holds because the $(\cos \theta ,\phi )$ two-dimensional distribution for the fully simulated unpolarised $\psi {(2S)}$ mesons is the same as the efficiency $\epsilon (\cos \theta ,\phi )$ up to a constant global factor.

**Fig. 3:**
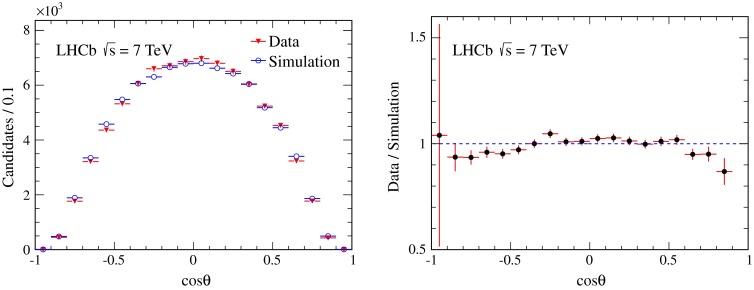
(*Left*) Distributions of $$\cos \theta $$ in the helicity frame for $${J /\psi }$$ mesons from $$B ^+ \rightarrow {J /\psi } K^+$$ decays in data (*filled triangles*) and in the simulated sample (*open circles*) and (*right*) their ratio after the weighting based on the $$B ^+ $$ and $${J /\psi }$$ kinematic properties

The angular efficiency is validated in data by using muons from $$B ^+ \!\rightarrow {J /\psi } K ^+ $$ decays. Due to angular momentum conservation, the $${J /\psi }$$ meson produced in this channel is longitudinally polarised in the $$B ^+ $$ meson rest frame. After reweighting the kinematic properties of the simulated $$B ^+ $$ and $${J /\psi }$$ mesons to reproduce the data, the remaining differences of the angular distributions between the reweighted simulation sample and the data are attributed to imperfections in the modelling of the detector response. Figure 3 compares the $$\cos \theta $$ distributions in data for $$B ^+ \!\rightarrow {J /\psi } K ^+ $$ candidates in the helicity frame with simulated data after reweighting. The efficiency for simulated events is overestimated for $${J /\psi }$$ candidates with $$|\cos \theta |>0.5$$, therefore it is corrected further as a function of $$p_\mu $$ and $$y_\mu $$, the momentum and the rapidity of the muon in the centre-of-mass frame of $$pp$$ collisions. A table of weights (corrections) in bins of $$p_\mu $$ and $$y_\mu $$ are determined by studying the two-dimensional $$(p_{\mu },y_{\mu })$$ distribution of $$B ^+ \!\rightarrow {J /\psi } K ^+ $$ candidates in data and simulation. The normalisation of Eq. 5 is calculated by assigning a weight to each candidate as the product of the weights for $$\mu ^+$$ and $$\mu ^-$$ depending on their $$p_\mu $$ and $$y_\mu $$ bins.

## Systematic uncertainties

Sources of systematic uncertainty are considered for each of the four observables $\lambda _\theta$, $\lambda _{\theta \phi }$, $\lambda _{\phi }$ and $\lambda _{\mathrm {inv}}$ in both the Collins-Soper and helicity frames. In the Collins-Soper frame, the overall systematic uncertainties are found to be comparable for each of these observables in most kinematic bins, while for the helicity frame the systematic uncertainties assigned to $\lambda _{\theta \phi }$ and $\lambda _{\phi }$ are typically a factor of 2–3 smaller than those estimated for $\lambda _\theta$ and $\lambda _{\mathrm {inv}}$. For each of the main sources of systematic uncertainty, Table 1 shows the range of values assigned over all kinematic bins, and their average. The total systematic uncertainties for each of the four observables can be found in Tables 2 and 3.

**Table 1:** Sources of systematic uncertainties on the polarisation parameter $\lambda _\theta$ in the helicity and Collins-Soper frames. For each type of uncertainty, the average and the range over all $\psi {(2S)}$ kinematic bins are shown

Source	Helicity frame	Collins-Soper frame
	Average (range)	Average (range)
Efficiency correction	0.055 (0.034–0.126)	0.035 (0.019–0.078)
Simulation sample size	0.034 (0.015–0.103)	0.023 (0.010–0.094)
Fit to mass distribution	0.008 (0.001–0.134)	0.007 (0.001–0.188)
$\psi {(2S)}$ kinematic modelling	0.018 (0.000–0.085)	0.016 (0.000–0.074)
$$b $$-hadron contamination	0.014 (0.002–0.035)	0.013 (0.002–0.063)

**Table 2:** Measured prompt $\psi {(2S)}$ polarisation parameters $\lambda _\theta$, $\lambda _{\theta \phi }$, $\lambda _{\phi }$ and $\lambda _{\mathrm {inv}}$ in bins of $p_\mathrm{T}$ and $y$ in the helicity frame. The first uncertainty is statistical and the is second systematic

$p_\mathrm{T}$ ($\mathrm {GeV\!/}c$)	$\lambda$	$2.0<y<2.5$	$2.5<y<3.0$	$3.0<y<3.5$	$3.5<y<4.0$	$4.0<y<4.5$
3.5–4	$\lambda _\theta$	$-$0.331 $\pm$ 0.174 $\pm$ 0.142	$-$0.055 $\pm$ 0.052 $\pm$ 0.056	0.028 $\pm$ 0.040 $\pm$ 0.046	0.008 $\pm$ 0.040 $\pm$ 0.050	$-$0.080 $\pm$ 0.063 $\pm$ 0.092
	$\lambda _{\theta \phi }$	$-$0.233 $\pm$ 0.076 $\pm$ 0.086	$-$0.172 $\pm$ 0.021 $\pm$ 0.026	$-$0.039 $\pm$ 0.020 $\pm$ 0.023	0.007 $\pm$ 0.021 $\pm$ 0.028	$-$0.048 $\pm$ 0.036 $\pm$ 0.049
	$\lambda _\phi$	$-$0.049 $\pm$ 0.036 $\pm$ 0.037	$-$0.039 $\pm$ 0.017 $\pm$ 0.024	$-$0.074 $\pm$ 0.018 $\pm$ 0.022	$-$0.081 $\pm$ 0.022 $\pm$ 0.027	$-$0.110 $\pm$ 0.043 $\pm$ 0.047
	$\lambda _\mathrm {inv}$	$-$0.456 $\pm$ 0.195 $\pm$ 0.160	$-$0.165 $\pm$ 0.063 $\pm$ 0.078	$-$0.180 $\pm$ 0.054 $\pm$ 0.063	$-$0.217 $\pm$ 0.057 $\pm$ 0.073	$-$0.371 $\pm$ 0.089 $\pm$ 0.114
4–5	$\lambda _\theta$	$-$0.194 $\pm$ 0.113 $\pm$ 0.113	0.007 $\pm$ 0.038 $\pm$ 0.052	$-$0.003 $\pm$ 0.028 $\pm$ 0.052	$-$0.026 $\pm$ 0.029 $\pm$ 0.052	0.007 $\pm$ 0.050 $\pm$ 0.095
	$\lambda _{\theta \phi }$	$-$0.238 $\pm$ 0.049 $\pm$ 0.053	$-$0.086 $\pm$ 0.016 $\pm$ 0.023	$-$0.026 $\pm$ 0.015 $\pm$ 0.021	0.003 $\pm$ 0.017 $\pm$ 0.025	0.023 $\pm$ 0.027 $\pm$ 0.043
	$\lambda _\phi$	$-$0.043 $\pm$ 0.023 $\pm$ 0.024	$-$0.082 $\pm$ 0.012 $\pm$ 0.014	$-$0.048 $\pm$ 0.012 $\pm$ 0.023	$-$0.087 $\pm$ 0.016 $\pm$ 0.025	$-$0.088 $\pm$ 0.033 $\pm$ 0.035
	$\lambda _\mathrm {inv}$	$-$0.309 $\pm$ 0.126 $\pm$ 0.120	$-$0.222 $\pm$ 0.045 $\pm$ 0.060	$-$0.140 $\pm$ 0.042 $\pm$ 0.057	$-$0.265 $\pm$ 0.044 $\pm$ 0.070	$-$0.237 $\pm$ 0.072 $\pm$ 0.102
5–7	$\lambda _\theta$	$-$0.198 $\pm$ 0.074 $\pm$ 0.091	0.083 $\pm$ 0.030 $\pm$ 0.051	0.003 $\pm$ 0.024 $\pm$ 0.039	$-$0.088 $\pm$ 0.024 $\pm$ 0.046	$-$0.189 $\pm$ 0.039 $\pm$ 0.092
	$\lambda _{\theta \phi }$	$-$0.164 $\pm$ 0.031 $\pm$ 0.039	$-$0.072 $\pm$ 0.013 $\pm$ 0.018	$-$0.026 $\pm$ 0.013 $\pm$ 0.020	0.002 $\pm$ 0.015 $\pm$ 0.026	0.044 $\pm$ 0.025 $\pm$ 0.051
	$\lambda _\phi$	$-$0.058 $\pm$ 0.014 $\pm$ 0.021	$-$0.046 $\pm$ 0.009 $\pm$ 0.013	$-$0.058 $\pm$ 0.009 $\pm$ 0.018	$-$0.038 $\pm$ 0.012 $\pm$ 0.019	$-$0.039 $\pm$ 0.025 $\pm$ 0.028
	$\lambda _\mathrm {inv}$	$-$0.352 $\pm$ 0.080 $\pm$ 0.094	$-$0.054 $\pm$ 0.039 $\pm$ 0.060	$-$0.162 $\pm$ 0.035 $\pm$ 0.054	$-$0.195 $\pm$ 0.040 $\pm$ 0.067	$-$0.294 $\pm$ 0.065 $\pm$ 0.105
7–10	$\lambda _\theta$	$-$0.142 $\pm$ 0.066 $\pm$ 0.079	$-$0.064 $\pm$ 0.034 $\pm$ 0.053	$-$0.001 $\pm$ 0.032 $\pm$ 0.051	$-$0.196 $\pm$ 0.033 $\pm$ 0.071	$-$0.159 $\pm$ 0.058 $\pm$ 0.118
	$\lambda _{\theta \phi }$	0.044 $\pm$ 0.028 $\pm$ 0.034	0.002 $\pm$ 0.014 $\pm$ 0.021	0.008 $\pm$ 0.016 $\pm$ 0.023	0.003 $\pm$ 0.019 $\pm$ 0.031	0.124 $\pm$ 0.037 $\pm$ 0.058
	$\lambda _\phi$	$-$0.036 $\pm$ 0.014 $\pm$ 0.017	$-$0.075 $\pm$ 0.010 $\pm$ 0.012	$-$0.088 $\pm$ 0.011 $\pm$ 0.012	$-$0.036 $\pm$ 0.014 $\pm$ 0.018	0.007 $\pm$ 0.030 $\pm$ 0.031
	$\lambda _\mathrm {inv}$	$-$0.241 $\pm$ 0.079 $\pm$ 0.095	$-$0.269 $\pm$ 0.043 $\pm$ 0.064	$-$0.245 $\pm$ 0.043 $\pm$ 0.062	$-$0.292 $\pm$ 0.052 $\pm$ 0.100	$-$0.140 $\pm$ 0.101 $\pm$ 0.138
10–15	$\lambda _\theta$	$-$0.137 $\pm$ 0.080 $\pm$ 0.123	$-$0.235 $\pm$ 0.047 $\pm$ 0.075	$-$0.258 $\pm$ 0.048 $\pm$ 0.073	$-$0.371 $\pm$ 0.059 $\pm$ 0.135	$-$0.706 $\pm$ 0.081 $\pm$ 0.161
	$\lambda _{\theta \phi }$	0.157 $\pm$ 0.034 $\pm$ 0.050	0.045 $\pm$ 0.020 $\pm$ 0.026	0.094 $\pm$ 0.023 $\pm$ 0.032	0.052 $\pm$ 0.031 $\pm$ 0.054	0.104 $\pm$ 0.059 $\pm$ 0.079
	$\lambda _\phi$	$-$0.014 $\pm$ 0.021 $\pm$ 0.022	$-$0.059 $\pm$ 0.017 $\pm$ 0.011	$-$0.074 $\pm$ 0.020 $\pm$ 0.014	$-$0.047 $\pm$ 0.027 $\pm$ 0.020	0.044 $\pm$ 0.053 $\pm$ 0.048
	$\lambda _\mathrm {inv}$	$-$0.176 $\pm$ 0.103 $\pm$ 0.164	$-$0.390 $\pm$ 0.062 $\pm$ 0.086	$-$0.446 $\pm$ 0.067 $\pm$ 0.096	$-$0.489 $\pm$ 0.089 $\pm$ 0.150	$-$0.601 $\pm$ 0.162 $\pm$ 0.136

**Table 3:** Measured prompt $\psi {(2S)}$ polarisation parameters $\lambda _\theta$, $\lambda _{\theta \phi }$, $\lambda _{\phi }$ and $\lambda _{\mathrm {inv}}$ in bins of $p_\mathrm{T}$ and $y$ in the Collins-Soper frame. The first uncertainty is statistical and the second is systematic

$p_\mathrm{T}$ ($\mathrm {GeV\!/}c$)	$\lambda$	$2.0<y<2.5$	$2.5<y<3.0$	$3.0<y<3.5$	$3.5<y<4.0$	$4.0<y<4.5$
3.5–4	$\lambda _\theta$	$-$0.457 $\pm$ 0.142 $\pm$ 0.144	$-$0.282 $\pm$ 0.026 $\pm$ 0.036	$-$0.105 $\pm$ 0.023 $\pm$ 0.031	$-$0.047 $\pm$ 0.028 $\pm$ 0.041	$-$0.168 $\pm$ 0.058 $\pm$ 0.076
	$\lambda _{\theta \phi }$	0.141 $\pm$ 0.088 $\pm$ 0.065	0.018 $\pm$ 0.027 $\pm$ 0.031	$-$0.043 $\pm$ 0.022 $\pm$ 0.027	$-$0.038 $\pm$ 0.024 $\pm$ 0.032	$-$0.010 $\pm$ 0.044 $\pm$ 0.059
	$\lambda _\phi$	$-$0.003 $\pm$ 0.039 $\pm$ 0.028	0.040 $\pm$ 0.020 $\pm$ 0.023	$-$0.027 $\pm$ 0.021 $\pm$ 0.024	$-$0.061 $\pm$ 0.023 $\pm$ 0.028	$-$0.076 $\pm$ 0.031 $\pm$ 0.045
	$\lambda _\mathrm {inv}$	$-$0.465 $\pm$ 0.194 $\pm$ 0.179	$-$0.169 $\pm$ 0.062 $\pm$ 0.068	$-$0.180 $\pm$ 0.054 $\pm$ 0.062	$-$0.218 $\pm$ 0.057 $\pm$ 0.076	$-$0.368 $\pm$ 0.089 $\pm$ 0.118
4–5	$\lambda _\theta$	$-$0.374 $\pm$ 0.077 $\pm$ 0.086	$-$0.192 $\pm$ 0.019 $\pm$ 0.032	$-$0.080 $\pm$ 0.017 $\pm$ 0.030	$-$0.075 $\pm$ 0.020 $\pm$ 0.035	$-$0.035 $\pm$ 0.042 $\pm$ 0.056
	$\lambda _{\theta \phi }$	0.103 $\pm$ 0.059 $\pm$ 0.062	$-$0.020 $\pm$ 0.019 $\pm$ 0.028	$-$0.010 $\pm$ 0.015 $\pm$ 0.035	$-$0.027 $\pm$ 0.017 $\pm$ 0.032	$-$0.047 $\pm$ 0.034 $\pm$ 0.057
	$\lambda _\phi$	0.032 $\pm$ 0.029 $\pm$ 0.027	$-$0.011 $\pm$ 0.017 $\pm$ 0.025	$-$0.021 $\pm$ 0.017 $\pm$ 0.024	$-$0.069 $\pm$ 0.019 $\pm$ 0.028	$-$0.073 $\pm$ 0.027 $\pm$ 0.041
	$\lambda _\mathrm {inv}$	$-$0.288 $\pm$ 0.125 $\pm$ 0.123	$-$0.221 $\pm$ 0.045 $\pm$ 0.061	$-$0.141 $\pm$ 0.042 $\pm$ 0.058	$-$0.264 $\pm$ 0.044 $\pm$ 0.071	$-$0.237 $\pm$ 0.072 $\pm$ 0.096
5–7	$\lambda _\theta$	$-$0.265 $\pm$ 0.040 $\pm$ 0.062	$-$0.147 $\pm$ 0.014 $\pm$ 0.024	$-$0.095 $\pm$ 0.015 $\pm$ 0.023	$-$0.029 $\pm$ 0.019 $\pm$ 0.030	0.038 $\pm$ 0.037 $\pm$ 0.067
	$\lambda _{\theta \phi }$	0.123 $\pm$ 0.041 $\pm$ 0.051	$-$0.022 $\pm$ 0.013 $\pm$ 0.026	$-$0.013 $\pm$ 0.011 $\pm$ 0.025	0.026 $\pm$ 0.013 $\pm$ 0.028	0.050 $\pm$ 0.029 $\pm$ 0.053
	$\lambda _\phi$	$-$0.024 $\pm$ 0.026 $\pm$ 0.032	0.033 $\pm$ 0.014 $\pm$ 0.024	$-$0.024 $\pm$ 0.015 $\pm$ 0.024	$-$0.060 $\pm$ 0.018 $\pm$ 0.030	$-$0.125 $\pm$ 0.029 $\pm$ 0.051
	$\lambda _\mathrm {inv}$	$-$0.330 $\pm$ 0.080 $\pm$ 0.098	$-$0.049 $\pm$ 0.040 $\pm$ 0.059	$-$0.163 $\pm$ 0.035 $\pm$ 0.056	$-$0.198 $\pm$ 0.040 $\pm$ 0.067	$-$0.299 $\pm$ 0.066 $\pm$ 0.106
7–10	$\lambda _\theta$	0.035 $\pm$ 0.039 $\pm$ 0.044	$-$0.078 $\pm$ 0.019 $\pm$ 0.028	$-$0.098 $\pm$ 0.020 $\pm$ 0.031	0.008 $\pm$ 0.028 $\pm$ 0.044	0.225 $\pm$ 0.061 $\pm$ 0.082
	$\lambda _{\theta \phi }$	0.006 $\pm$ 0.038 $\pm$ 0.046	$-$0.002 $\pm$ 0.014 $\pm$ 0.023	$-$0.034 $\pm$ 0.013 $\pm$ 0.021	0.065 $\pm$ 0.017 $\pm$ 0.031	$-$0.017 $\pm$ 0.040 $\pm$ 0.058
	$\lambda _\phi$	$-$0.096 $\pm$ 0.032 $\pm$ 0.037	$-$0.070 $\pm$ 0.019 $\pm$ 0.031	$-$0.053 $\pm$ 0.019 $\pm$ 0.031	$-$0.111 $\pm$ 0.025 $\pm$ 0.040	$-$0.131 $\pm$ 0.045 $\pm$ 0.065
	$\lambda _\mathrm {inv}$	$-$0.230 $\pm$ 0.079 $\pm$ 0.093	$-$0.269 $\pm$ 0.043 $\pm$ 0.066	$-$0.244 $\pm$ 0.043 $\pm$ 0.062	$-$0.293 $\pm$ 0.052 $\pm$ 0.081	$-$0.149 $\pm$ 0.101 $\pm$ 0.137
10–15	$\lambda _\theta$	0.163 $\pm$ 0.055 $\pm$ 0.055	0.042 $\pm$ 0.037 $\pm$ 0.042	0.087 $\pm$ 0.045 $\pm$ 0.052	0.138 $\pm$ 0.063 $\pm$ 0.089	0.675 $\pm$ 0.175 $\pm$ 0.222
	$\lambda _{\theta \phi }$	$-$0.103 $\pm$ 0.043 $\pm$ 0.065	0.015 $\pm$ 0.022 $\pm$ 0.026	$-$0.024 $\pm$ 0.024 $\pm$ 0.023	0.062 $\pm$ 0.034 $\pm$ 0.046	0.221 $\pm$ 0.090 $\pm$ 0.075
	$\lambda _\phi$	$-$0.117 $\pm$ 0.044 $\pm$ 0.068	$-$0.163 $\pm$ 0.032 $\pm$ 0.045	$-$0.211 $\pm$ 0.036 $\pm$ 0.045	$-$0.251 $\pm$ 0.050 $\pm$ 0.080	$-$0.539 $\pm$ 0.117 $\pm$ 0.133
	$\lambda _\mathrm {inv}$	$-$0.168 $\pm$ 0.103 $\pm$ 0.162	$-$0.385 $\pm$ 0.063 $\pm$ 0.088	$-$0.450 $\pm$ 0.067 $\pm$ 0.086	$-$0.492 $\pm$ 0.089 $\pm$ 0.149	$-$0.613 $\pm$ 0.161 $\pm$ 0.130

The dominant systematic uncertainty is due to the size of the $B ^+ \!\rightarrow {J /\psi } K ^+$ control sample. This leads to non-negligible statistical uncertainties in the correction factors that are applied to simulated events in bins of $p_\mu$ and $y_\mu$. The uncertainty on a given correction factor is estimated by varying it by one standard deviation of its statistical uncertainty, while keeping all other factors at their central values. The polarisation parameters are recalculated and the change relative to their default values is considered as the contribution from this factor to the systematic uncertainty. This procedure is repeated for all bins of $p_\mu$ and $y_\mu$, and the sum in quadrature of all these independent contributions is taken as the total systematic uncertainty.

The limited size of the sample of simulated events introduces an uncertainty on the normalisation $N(\lambda _\theta , \lambda _{\theta \phi },\lambda _\phi )$, and this uncertainty is propagated to the polarisation parameters.

The uncertainty of the *sWeight*  of each candidate used for the background subtraction is a source of uncertainty on the polarisation parameters. The effect is studied by comparing the default polarisation parameters with those obtained when varying the definition of the models used to fit the mass distributions and re-evaluating the *sWeight* for each candidate. Several alternative fitting models are studied, including an exponential function for the background mass distribution, only one CB function for the signal mass distribution, or shapes for signal and background mass distributions fixed to those obtained from fits to the mass distributions in sub-regions of the $(\cos \theta ,\phi )$ distribution space. The largest variation with respect to the default result is assigned as the systematic uncertainty.

In each kinematic bin, discrepancies between data and simulation in the $\psi {(2S)}$ $p_\mathrm{T}$ and $y$ distributions introduce an additional uncertainty. This is evaluated by comparing the default polarisation results with those determined after the $\psi {(2S)}$ kinematic distribution in the simulation is weighted to that in data. The difference between the two results is quoted as a systematic uncertainty contribution.

The uncertainty due to the contamination of $\psi {(2S)}$ candidates from $b$-hadron decays ($3~\%$) is determined by relaxing the $S_\tau$ selection and studying the variations of the polarisation parameters.

With the exception of the effects due to the differences in the $\psi {(2S)}$ kinematic spectrum and the size of the sample of simulated events, correlations are expected among $\psi {(2S)}$ kinematic bins. The correlation between these systematic uncertainties in adjacent bins could be as large as 50 %, as the final state muons may have similar momentum and rapidity. For each kinematic bin, the total systematic uncertainty is calculated as the quadratic sum of the various sources of systematic uncertainties assuming no correlation within each kinematic bin.

## Results

**Fig. 4:**
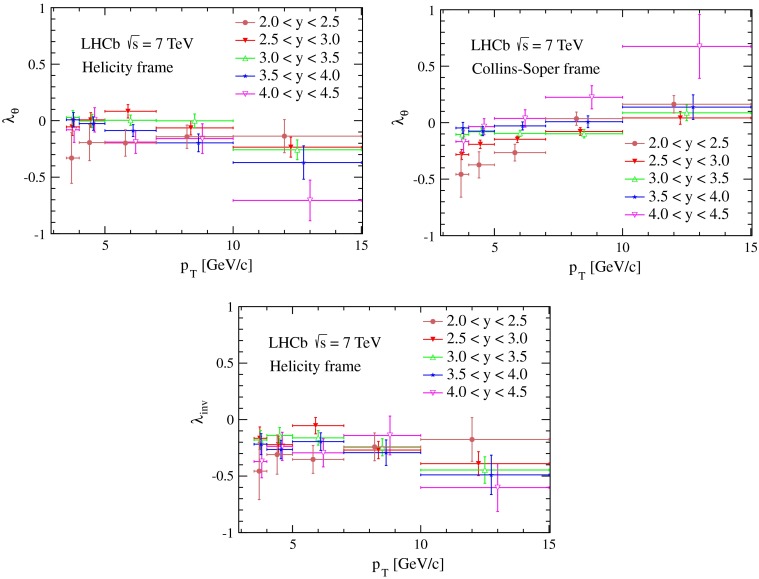
Polarisation parameters for prompt $\psi {(2S)}$ mesons as a function of $p_\mathrm{T}$, in five rapidity intervals, (*top left*) $\lambda _\theta$ and (*bottom*) $\lambda _{\mathrm {inv}}$, measured in the helicity frame, and (*top right*) $\lambda _\theta$ in the Collins-Soper frame. The uncertainties on data points are the sum in quadrature of statistical and systematic uncertainties. The *horizontal bars* represent the width of the $p_\mathrm{T}$ bins for the $\psi {(2S)}$ meson. The data points for each rapidity interval are displaced horizontally to improve visibility

The results for the polarisation parameters $\lambda _\theta$, $\lambda _{\theta \phi }$, $\lambda _{\phi }$ and $\lambda _{\mathrm {inv}}$, and their uncertainties, in each $p_\mathrm{T}$ and $y$ bin of the prompt $\psi {(2S)}$ meson sample, are reported in Tables 2 and 3 for the helicity and the Collins-Soper frames, respectively. The systematic uncertainties are similar in size to the statistical uncertainties. The parameters $\lambda _\theta$ and $\lambda _{\mathrm {inv}}$ are also shown in Fig. 4 as functions of the $p_\mathrm{T}$ of the $\psi {(2S)}$ mesons, for different $y$ bins.

The frame-invariant polarisation parameter $\lambda _{\mathrm {inv}}$ is consistent with a negative polarisation with no strong dependence on the $p_\mathrm{T}$ and $y$ of the $\psi {(2S)}$ meson. The values and uncertainties of $\lambda _{\mathrm {inv}}$ that are measured in the helicity and the Collins-Soper frames are in good agreement with each other, with differences much smaller than the statistical uncertainties. In the Collins-Soper frame, $\lambda _\theta$ takes small negative values especially in the low-$p_\mathrm{T}$ region and increases with $p_\mathrm{T}$. This trend is more significant for the extreme $y$ bins. In the helicity frame, the polarisation parameter $\lambda _\theta$ is consistent with zero, with no significant dependence on $p_\mathrm{T}$ or $y$ of the $\psi {(2S)}$ meson. The polarisation parameters $\lambda _{\theta \phi }$ and $\lambda _{\phi }$ are consistent with zero in both the helicity and Collins-Soper frames, and their absolute values are below 0.1 for most of the kinematic bins.

**Fig. 5:**
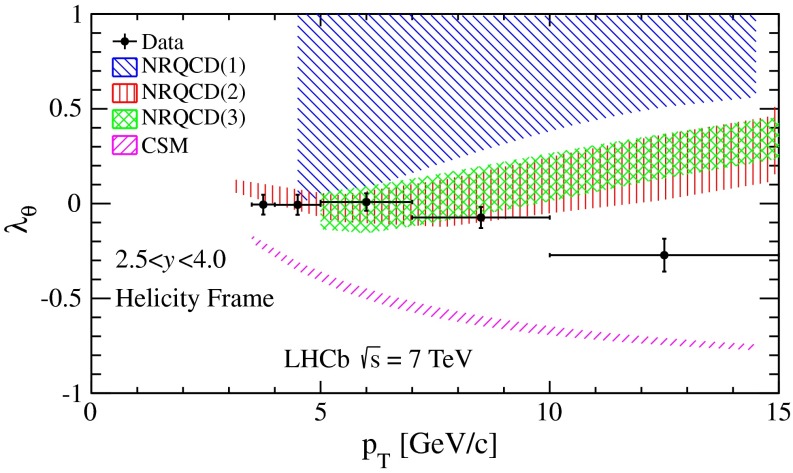
Polarisation parameter $\lambda _\theta$ of the prompt $\psi {(2S)}$ meson in the helicity frame as a function of $p_\mathrm{T}$, in the rapidity range $2.5<y<4$. The predictions of NLO CSM [39] and three NLO NRQCD models (1) [39], (2) [40] and (3) [41] are also shown. Uncertainties on data are the sum in quadrature of the statistical and systematic uncertainties. The *horizontal bars* represent the width of $p_\mathrm{T}$ bins for the $\psi {(2S)}$ meson

In Fig. 5, the measured values of $\lambda _\theta$ in the helicity frame as a function of $p_\mathrm{T}$ of the $\psi {(2S)}$ meson, integrating over the rapidity range $2.5<y<4.0$, are compared with the predictions of the CSM [39] and NRQCD [39–41] models at NLO. Our results disfavour the CSM calculations, in which the $\psi {(2S)}$ meson is significantly longitudinally polarised. The three NRQCD calculations in Refs. [39–41], which use different selections of experimental data to determine the non-perturbative matrix elements, provide a good description of our measurements in the low-$p_\mathrm{T}$ region. However, the prediction of increasing polarisation with $p_\mathrm{T}$ in these models is not supported by the LHCb data.

## Conclusion

The polarisation of prompt $\psi {(2S)}$ mesons is measured as a function of the $\psi {(2S)}$ $p_\mathrm{T}$ and $y$ in the range $3.5<$ $p_\mathrm{T} <15{\mathrm {\,GeV\!/}c}$ and $2.0<y<4.5$, in $pp$ collisions at $\sqrt{s}= 7\,\mathrm{TeV}$. The analysis is performed using data corresponding to an integrated luminosity of 1.0 $\,\text{ fb }^{-1}$, collected by the LHCb experiment in 2011. The polarisation parameters $\lambda _\theta$, $\lambda _{\theta \phi }$, $\lambda _{\phi }$ and $\lambda _{\mathrm {inv}}$ are determined in the helicity and Collins-Soper frames by studying the angular distribution of the two muons produced in the $\psi {(2S)} \!\rightarrow \mu ^+ \mu ^-$ decay.

The frame-independent observable $\lambda _{\mathrm {inv}}$ is consistent with a negative polarisation. The measured values of $\lambda _{\theta \phi }$ and $\lambda _{\phi }$ are small over the accessible kinematic range. The $\lambda _\theta$ distribution in the helicity frame shows that the $\psi {(2S)}$ meson exhibits neither large transverse nor longitudinal polarisation. Although a direct comparison with previous measurements by CMS and CDF is not possible due to the different kinematic ranges, all results disfavour large polarisation in the high-$p_\mathrm{T}$ region. The prompt $\psi {(2S)}$ polarisation measured at LHCb disagrees with the CSM predictions both in the size of the polarisation parameters and the $p_\mathrm{T}$ dependence. While the NRQCD models provide a good description of the LHCb data in the low-$p_\mathrm{T}$ region, the predicted transverse polarisation at high-$p_\mathrm{T}$ is not observed.

## Appendix

See Tables 2 and 3.

## Footnotes

1 For a $\psi {(2S)}$ meson in a pure spin state the three polarisation parameters cannot vanish simultaneously.
